# Non-Destructive Lichen Biomass Estimation in Northwestern Alaska: A Comparison of Methods

**DOI:** 10.1371/journal.pone.0103739

**Published:** 2014-07-31

**Authors:** Abbey Rosso, Peter Neitlich, Robert J. Smith

**Affiliations:** 1 National Park Service, Winthrop, Washington, United States of America; 2 Department of Botany and Plant Pathology, Oregon State University, Corvallis, Oregon, United States of America; Field Museum of Natural History, United States of America

## Abstract

Terrestrial lichen biomass is an important indicator of forage availability for caribou in northern regions, and can indicate vegetation shifts due to climate change, air pollution or changes in vascular plant community structure. Techniques for estimating lichen biomass have traditionally required destructive harvesting that is painstaking and impractical, so we developed models to estimate biomass from relatively simple cover and height measurements. We measured cover and height of forage lichens (including single-taxon and multi-taxa “community” samples, *n* = 144) at 73 sites on the Seward Peninsula of northwestern Alaska, and harvested lichen biomass from the same plots. We assessed biomass-to-volume relationships using zero-intercept regressions, and compared differences among two non-destructive cover estimation methods (ocular vs. point count), among four landcover types in two ecoregions, and among single-taxon vs. multi-taxa samples. Additionally, we explored the feasibility of using lichen height (instead of volume) as a predictor of stand-level biomass. Although lichen taxa exhibited unique biomass and bulk density responses that varied significantly by growth form, we found that single-taxon sampling consistently under-estimated true biomass and was constrained by the need for taxonomic experts. We also found that the point count method provided little to no improvement over ocular methods, despite increased effort. Estimated biomass of lichen-dominated communities (mean lichen cover: 84.9±1.4%) using multi-taxa, ocular methods differed only nominally among landcover types within ecoregions (range: 822 to 1418 g m^−2^). Height alone was a poor predictor of lichen biomass and should always be weighted by cover abundance. We conclude that the multi-taxa (whole-community) approach, when paired with ocular estimates, is the most reasonable and practical method for estimating lichen biomass at landscape scales in northwest Alaska.

## Introduction

Lichen biomass is an important indicator of grazing impact and the availability of winter forage for caribou, reindeer, muskox, and other animals in northern regions [1], [2]. In northwestern Alaska, these animals rely on forage lichens including many species of *Cladonia* (“reindeer lichen”, previously *Cladina*), *Alectoria*, *Bryocaulon*, *Bryoria*, and *Cetraria* [3]. Lichens provide the main winter sustenance for the Western Arctic Caribou Herd, which is one of the largest caribou herds in North America and is important in the subsistence economy of native Alaskans [1]. Aside from utility as wildlife forage, terrestrial lichen biomass can also be used as a vegetation monitoring metric to assess the impact of disturbances such as fire [3]–[5], climate change [6], [7] and air pollution [8], [9]. However, estimating lichen biomass by destructive sampling is very time consuming and does not allow for assessment of the same area over time. Researchers studying epiphytic lichens have previously approached this problem in places such as Norway [10], China [11], British Columbia [12] and the U.S. Pacific Northwest [13] by developing regression equations, yet very few have estimated biomass for ground-dwelling, terrestrial lichens.

Biomass estimation requires accurate volumetric measurements for lichen height and area cover. The method by which cover is measured represents a trade-off between the potential bias of estimates (if estimated subjectively) and the potential to under-represent rare or patchily distributed taxa (if measured quantitatively) [14]. Point-count estimates have the potential to be less biased given a high enough density of points [15], while ocular cover estimates can assess larger areas rapidly and can integrate patchiness. Therefore, ocular and point count methods must be comparatively assessed for accuracy and for their influence on biomass estimates.

Climate and vegetation type are important predictors of lichen biomass [16] and should be accounted for in landscape-level assessments. The landscapes of northwestern Alaska span several climatic ecoregions and a wide variety of vegetation and landcover types including wetlands, spruce woodlands, and tundra dominated by lichens, low shrubs or graminoids [17], [18]. Within these landcover types, there are strong gradients of substrate pH and vascular vegetation physiognomy that influence lichen community composition [19]. Because species differ in growth forms and levels of dominance, it could be expected that variability in lichen biomass and bulk density would be associated with distinctive landcover types.

Lichens in northwestern Alaska occur in very diverse, highly mixed assemblages typified by high species richness [3], [19]. Yet, most estimates of terrestrial lichen biomass completed elsewhere have been limited to only a single [5] or a very restricted number of species [2], [20], mainly focusing on the critical forage lichen genus *Cladonia*. Some workers [20] have suggested that in diverse lichen communities, the most rapid and effective way to estimate biomass would be to integrate estimates over entire communities rather than parsing out unique biomass relationships among individual taxa. Whole-community surveys [21] are also the most realistic way to monitor biomass across landscapes that have heterogeneous or patchy distributions of a large number of species, or for landscapes that have large species turnover (beta-diversity) among sites.

This study explored the feasibility of using height and cover measurements to estimate terrestrial lichen biomass in northwestern Alaska. Our inferences focused mainly on forage lichens (including *Alectoria*, *Bryocaulon*, *Cetraria* and *Cladonia* spp.) considered both as individual taxa and as the dominant members of aggregate, multi-taxa lichen communities. Here, we extended the range of species to include multi-taxa “community” samples that integrated all observed species, and we presented regression slope estimates to use as conversion factors applicable to northwestern Alaska. Our primary objectives in this study were: 1) to estimate the slope of zero-intercept linear regressions for biomass vs. volume; 2) to examine model fit between single-taxon vs. multi-taxa sampling techniques; 3) to examine model fit between ocular vs. point count cover estimation techniques; 4) to determine whether observed and predicted biomass in multi-taxa communities differed significantly among landcover types within ecoregions; and 5) to examine whether height or cover by themselves could each be good predictors of lichen biomass. We also place our results in the context of previous studies and make recommendations for best survey practices.

## Methods

### Ethics Statement

Field sampling was conducted on public lands administered by the US Bureau of Land Management by permission of that agency. We complied with all national and international rules regarding ethics; the research did not involve measurements on humans or animals. Plant material collected for this study was sampled on a very limited scale and therefore had negligible effects on landscapes. We declare no commercial interests or conflicts of interest.

### Field Sampling

Field data are available as Dataset S1. We measured lichen height, cover and biomass (both for single taxa and for all taxa aggregated) among two different ecoregions [22] and four landcover types on the Seward Peninsula, northwestern Alaska (Table 1). Although both ecoregions converge on the Seward Peninsula, they differ sharply in rainfall and climate [23]. The first ecoregion, the Northern Seward Peninsula/Kotzebue Sound Lowlands (hereafter, “Kotzebue Lowlands”), has a Chukchi Sea-influenced and more continental climate with mean annual precipitation of 250–500 mm. The second ecoregion, the Southern Seward Peninsula/Bering Sea Coast (hereafter “Bering Sea Coast”) is moist with mean annual precipitation ranging from 400–800 mm. Within each ecoregion we sampled four landcover types [17] which formed the basis of long-term lichen monitoring in the Bering Land Bridge National Preserve [24]; these were: Dwarf-Shrub and Lichen Dominated (L), Mesic/Dry Herbaceous (M), Open Low Shrub – Dwarf Birch/Ericaceous (P), and Sparse Vegetation (S) [17]. While there was a newer landcover classification available at the time of sampling, we chose an older data set compatible with NPS's Arctic Network's long-term lichen monitoring studies [24].

**Table 1:** Number of lichen samples among habitats and ecoregions of northwestern Alaska for all sample types.

Land cover type	Ecoregion	Sample type	*Alectoria*	*Bryocaulon*	*Cetraria*	*Cladonia*	*C. stellaris*	Total
L	N	Single-taxon	4	7	1	3	4	19
		Multi-taxa	2	2	1	12	1	18
	S	Single-taxon	0	0	0	0	0	0
		Multi-taxa	0	0	0	0	0	0
M	N	Single-taxon	0	0	5	2	0	7
		Multi-taxa	0	0	0	6	1	7
	S	Single-taxon	0	0	2	3	0	5
		Multi-taxa	0	0	0	2	0	2
P	N	Single-taxon	0	0	7	2	0	9
		Multi-taxa	0	0	0	11	0	11
	S	Single-taxon	3	0	6	3	0	12
		Multi-taxa	0	0	2	14	0	16
S	N	Single-taxon	7	7	0	3	2	19
		Multi-taxa	1	5	0	8	1	15
	S	Single-taxon	0	2	0	0	0	2
		Multi-taxa	1	0	0	1	0	2
Total:			18	23	24	70	9	144

Habitat type codes [17]: L = Dwarf Shrub-Lichen Dominated; M = Mesic/Dry Herbaceous; P = Open Low Shrub Dwarf Birch/Ericaceous; S = Sparse Vegetation. “*Cladonia*” includes all species except *C. stellaris*. Ecoregion codes [18]: N = Northern Seward Peninsula/Kotzebue Sound Lowlands (Kotzebue Lowlands) and S = Southern Seward Peninsula/Bering Sea Coast (Bering Coast).

In the study area, we assigned plot locations non-randomly. For single-taxon sampling, we targeted sites with continuous coverage of a dominant forage lichen species. For multi-taxa sampling, we targeted sites with continuous coverage of lichens in general, avoiding non-lichen vegetation. Because multi-taxa communities were often dominated by high proportions of forage lichens (*Alectoria* spp., *Bryocaulon divergens, Cetraria* spp., or branched-fruticose *Cladonia*), we recorded the dominant lichen taxon within each community to facilitate later comparison with single-taxa samples, and also recorded the dominant vascular vegetation type for landcover classification according to [17].

On the ground at each sampling location, we placed a quadrat frame made of PVC pipe (internal area 25×25 cm) divided into twenty-five cells (5×5 cm). In the center of each cell, we measured the height of lichens (if present) using a narrow, ruled metal rod (3 mm diameter) lowered to the base of the lichen until firm resistance at the duff/soil layer was encountered, but not pushing into layers below. We calculated lichen height as the average of the twenty-five points within each plot (including “no-hit” zero values) because we wanted to facilitate adaptation to other techniques that might include many lichen absences (e.g., other point count or line intercept surveys). For single-taxon samples, we recorded only the height of the target lichen, while for multi-taxa samples we also recorded the identity of the lichen species and visually estimated its cover to the nearest one percent. Based on ground surface similarity of growth form (i.e., without dislodging the lichen) and bulk density, species found in single-taxon plots were aggregated to the genus level if several were present in the same sample. For example, *Alectoria ochroleuca* was grouped with *A. nigricans*, and *Cetraria laevigata* was grouped with *C. islandica*. Morphologically similar *Cladonia* species (*C. arbuscula and C. mitis*; *C. rangiferina* and *C. stygia*) were also grouped, although we measured *C. stellaris* separately because it had apparently different bulk density and a morphology that was readily recognizable in the field.

After recording height and cover, lichens were misted with water, then manually harvested by cutting vertically along the edge of each quadrat and gently separating intact mats from the ground. We removed non-lichen plant parts, non-target species, obviously dead or decaying lichen parts, and any dirt, gravel or extraneous debris, then placed samples in dry paper bags for transport to the lab. Each sample was completely oven-dried at 80°C until reaching a stable, unchanging mass (minimum 8 hours, no more than 12 hours). Note that this is considerably less drying time than the 76 hours used in a similar study [20]. We weighed each sample with a digital scale (precision ± 0.01 g) within just a few minutes of removal from the drying oven to prevent moisture uptake that might bias dry mass estimates. This also differs from [20], in which lichens were allowed to cool to room temperature before weighing.

### Calculations

Our goal was to assess the relationship between biomass and lichen volume (calculated as the product of cover×height). We used two methods to calculate volume; the first was based on the ocular (visual) method of estimating cover described above, and the second was based on the point count estimation of cover. For the point count method, we estimated percentage cover by dividing the number of lichen presences by the total number of points (twenty-five) in each plot. For both methods, we multiplied percentage cover by the area of the plot to convert all measurements to cubic centimeters.

We regressed lichen biomass on volume (or height or cover) using ordinary least squares regression with forced zero intercepts implemented in R version 3.0.1 (R Core Development Team 2013) and compared model slope parameters. Although weighted regressions have been used elsewhere [20] to estimate forage lichen biomass, examination of model residuals (not reported here) suggested this was not necessary given our data. Because goodness-of-fit *R^2^* statistics are artificially inflated and are essentially meaningless for zero-intercept models, we instead report confidence intervals for each slope, and compare models using three metrics: percent difference in slope, Bayesian Information Criterion (*BIC*) [25] and log-likelihood ratio (*LLR*) tests [26]. The latter two are likelihood-based methods that do not attempt to assess whether two model parameters differ more than expected by chance (as would a probability-based *F*-test, for example), but instead they provide a relative measure of how much better or worse each model fits, given the data at hand. For our application, this is more useful than *F*-tests because the sign and magnitude of difference between two methods provides more information than simply whether or not any such difference occurs.

To determine whether mean observed and predicted biomass in multi-taxa “communities” differed significantly among landcover types within ecoregions, we used nested analysis-of-variance *F*-tests (landcover type nested within ecoregions), accompanied by Tukey's Honest Significant Difference test for pairwise comparisons; this procedure used only the multi-taxa data to simulate realistic conditions.

## Results

### Biomass vs. Volume Slope

**Figure 1:**
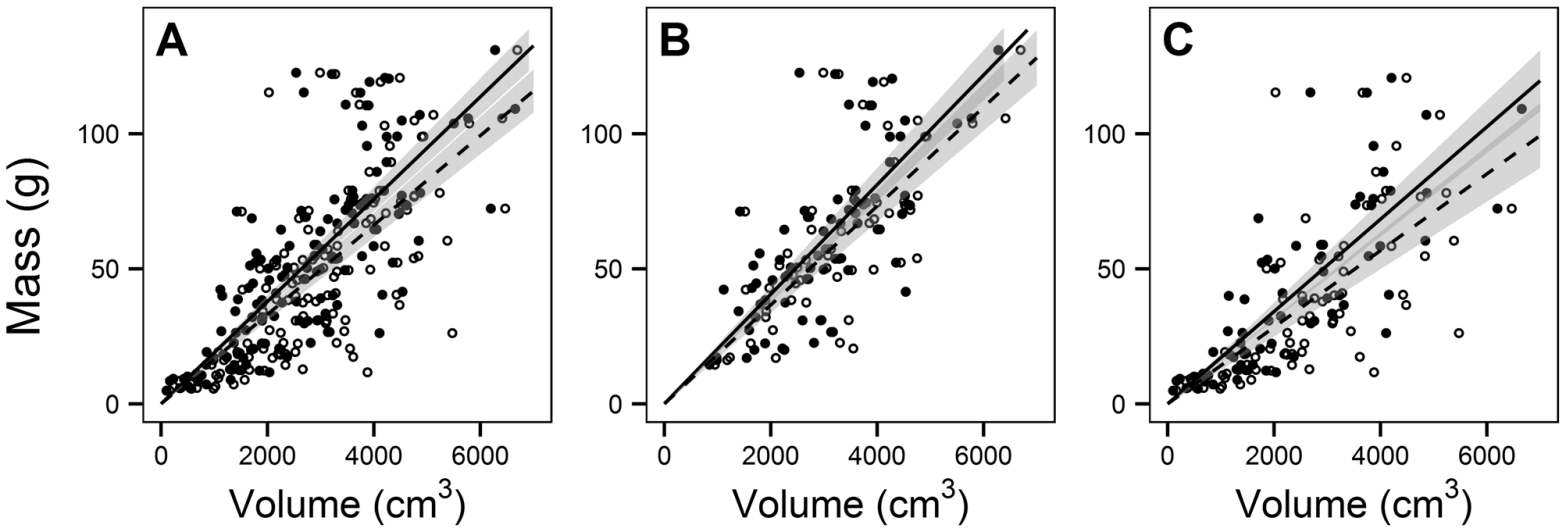
Biomass-volume relations using multiple methods. Shown are the ocular cover method (filled circles and solid lines) and the point count method (open circles and dotted lines), for all sample types combined (Fig. 1A), multi-taxa samples (Fig. 1B), and single-taxon samples (Fig. 1C). Lines indicate fitted model slopes (see Table 2 for estimates), while grey polygons indicate 95% confidence intervals.

From the regression of biomass on volume, estimates of the slope ranged from 0.0143 to 0.0203 g cm^−3^ for lichen communities, depending on the method used (Table 2 and Fig. 1A). Individual taxa ranged from 0.0056 g cm^−3^ (*Alectoria*) to 0.0221 g cm^−3^ (*Cladonia stellaris*). All slopes differed significantly from zero (*p*<0.05 from *t*-test of linear slope coefficients). For both observed mass and bulk density, mean values differed among all taxon groups (*F*-tests *p*<0.0001) except the pairwise comparisons among *Bryocaulon –Cetraria* and *Cladonia–C. stellaris* (for all others, *p*<0.05 from Tukey test).

**Table 2:** Estimated parameters and goodness-of-fit statistics for fitted models of forage lichen biomass in northwestern Alaska.

Method	Sample type	Taxon	Slope coefficient (g cm^−3^)			Bayesian Information Criterion (*BIC*)			Log-likelihood and *LLR*		
			Ocular	Pt count	Difference	Ocular	Pt count	Difference	Ocular	Pt count	*LLR*
Combined	All	All	0.0176	-	-	2607.35	-	-	−1298.01	-	-
Either	All	All	0.0189	0.0165	−12.8%	1285.43	1319.60	2.7%	−637.75	−654.83	−34.17
Either	Multi-taxa	All	0.0203	0.0183	−9.8%	641.14	647.12	0.9%	−316.31	−319.30	−5.98
Either	Single-taxa	All	0.0171	0.0143	−16.4%	642.61	666.68	3.7%	−317.01	−329.05	−24.08
Either	Multi-taxa	*Alectoria*	0.0106	0.0098	−7.2%	35.27	33.63	−4.7%	−16.25	−15.43	1.64
Either	Multi-taxa	*Bryocaulon*	0.0160	0.0154	−3.8%	54.72	53.66	−1.9%	−25.41	−24.88	1.06
Either	Multi-taxa	*Cetraria*	0.0177	0.0135	−23.4%	19.87	16.89	−15.0%	−8.84	−7.34	2.98
Either	Multi-taxa	*Cladonia*	0.0207	0.0186	−10.1%	481.10	486.65	1.2%	−236.56	−239.34	−5.56
Either	Multi-taxa	*C. stellaris*	0.0299	0.0288	−3.6%	21.56	16.35	−24.2%	−9.68	−7.08	5.21
Either	Single-taxa	*Alectoria*	0.0083	0.0056	−33.3%	93.63	95.56	2.1%	−44.18	−45.14	−1.93
Either	Single-taxa	*Bryocaulon*	0.0140	0.0120	−14.6%	128.78	123.45	−4.1%	−61.62	−58.95	5.33
Either	Single-taxa	*Cetraria*	0.0141	0.0105	−25.5%	157.81	153.10	−3.0%	−75.86	−73.51	4.71
Either	Single-taxa	*Cladonia*	0.0197	0.0184	−6.6%	155.42	158.15	1.8%	−74.94	−76.30	−2.73
Either	Single-taxa	*C. stellaris*	0.0221	0.0208	−6.1%	55.66	56.34	1.2%	−26.04	−26.38	−0.68
Height only	Multi-taxa	All	-	11.05 (g cm^−1^)	-	-	653.31	-	-	−322.4	-
Either, cover only	Multi-taxa	All	0.1188 (g cm^−2^)	0.1049 (g cm^−2^)	11.7%	672.70	682.46	1.5%	−332.1	−337.0	−9.76

“Method” column summarizes either ocular or point count methods of estimating volume, both of these, or height only. “Slope coefficient” is in units of g cm^−3^ unless otherwise noted, and is a measure of lichen mat bulk density; uncertainty of each estimate is presented graphically as 95% confidence intervals in Fig. 3. “Difference” between the ocular and point count method is expressed as a proportion of the ocular method. Model goodness-of-fit was determined by likelihood-based methods: better models have values closer to zero for Bayesian Information Criterion (*BIC*) values and greater log-likelihood values. Log-likelihood ratios (*LLR*) were calculated holding ocular method as the null hypothesis, therefore, more strongly negative values indicate better fit for models using the ocular method. Each *LLR* test was significant (*p*<0.0001).

### Single-taxon vs. Multi-taxa Sampling

The single-taxon method consistently underestimated biomass relative to the multi-taxa method (Fig. 1B vs. 1C). Model fit was always better for the multi-taxa method (Table 2), which in all cases also had less variation in biomass than the single-taxon method (Table 3).

**Table 3:** Observed and predicted lichen biomass (g m^−2^) for each sample type and landcover type in northwestern Alaska.

.		Observed	Volume (ocular)		Volume (pt ct)		Height only		Cover only (ocular)		Cover only (pt ct)	
Sample type	Land cover type	Mean	Mean	Deviation	Mean	Deviation	Mean	Deviation	Mean	Deviation	Mean	Deviation
Multi-taxa	L	911.9 (102.1)	943.9 (69.0)	3.5%	916.2 (61.3)	0.5%	907.3 (56.9)	−0.5%	1068.1 (25.0)	17.1%	1021.3 (14.4)	12.0%
Multi-taxa	M	1508.6 (113.1)	1418.5 (104.9)	−6.0%	1379.7 (92.7)	−8.5%	1357.7 (93.6)	−10.0%	1080 (21.9)	−28.4%	1030.7 (10.2)	−31.7%
Multi-taxa	P	789.6 (77.0)	822 (75.8)	4.1%	899.2 (77.5)	13.9%	932.7 (71.4)	18.1%	893.4 (22.3)	13.1%	960.7 (17.3)	21.7%
Multi-taxa	S	1124.1 (118.5)	1021.4 (76.0)	−9.1%	978.6 (59.1)	−12.9%	957.1 (55.6)	−14.9%	1089.7 (28.7)	−3.1%	1034.5 (10.1)	−8.0%
Multi-taxa	All	991.9 (56.8)	976.3 (45.5)	1.6%	983.4 (41.7)	0.9%	986.0 (38.8)	0.6%	1008.3 (16.7)	−1.7%	1002.6 (8.8)	−1.1%
Single taxon	L	671.3 (110.5)	653.7 (86.9)	−2.6%	710.1 (68.9)	5.8%	-	-	-	-	-	-
Single taxon	M	809.9 (192.5)	665.2 (126.7)	−17.9%	651.9 (104.7)	−19.5%	-	-	-	-	-	-
Single taxon	P	435.0 (88.4)	435.0 (89.5)	0.0%	447.0 (76.8)	2.8%	-	-	-	-	-	-
Single taxon	S	676.2 (99.9)	764.5 (74.6)	13.1%	739.3 (62.7)	9.3%	-	-	-	-	-	-
Single-taxon	All	627.5 (58.2)	624.5 (47.0)	0.5%	633.2 (40.0)	−0.9%	-	-	-	-	-	-

Mean lichen biomass, estimated using the “All Taxa” regression slope coefficients from Table 2 and parceled out by landcover type, is reported with standard errors (in parentheses) and mean deviation from observed biomass. Mean deviation is interpreted as measurement error. Landcover type codes: L = Dwarf Shrub-Lichen Dominated; M = Mesic/Dry Herbaceous; P = Open Low Shrub Dwarf Birch/Ericaceous; S = Sparse Vegetation. Caution: these biomass values were based on average quadrat cover >80% and should *not* be considered an inference about areas >1 m^2^ (which could have erratic cover).

### Ocular vs. Point Count

**Figure 2:**
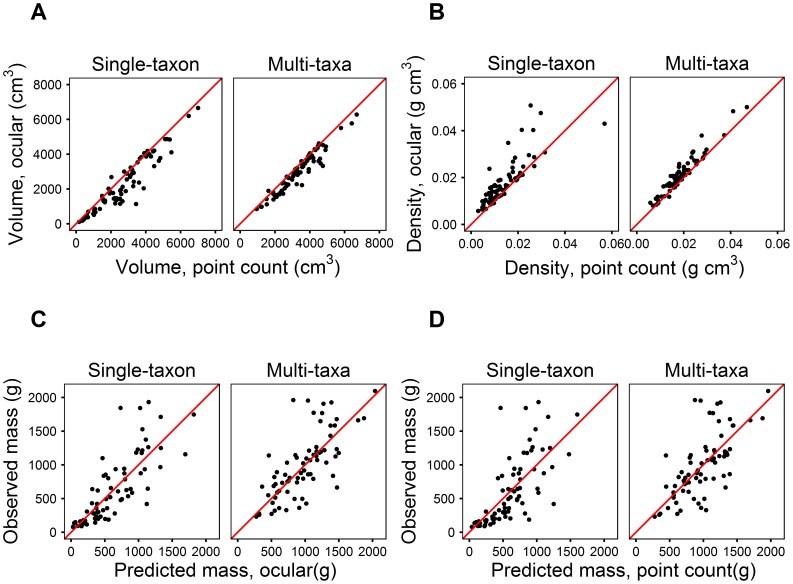
Relative comparisons of bias among two methods. Comparisons are between ocular vs. point count methods for estimates of volume (A) and estimates of bulk density (B); relative comparisons for observed vs. predicted lichen biomass for the ocular method (C) and the point count method (D). Each red line is a hypothetical 1∶1 isoline, where deviations from this line indicate magnitude of differences.

**Figure 3:**
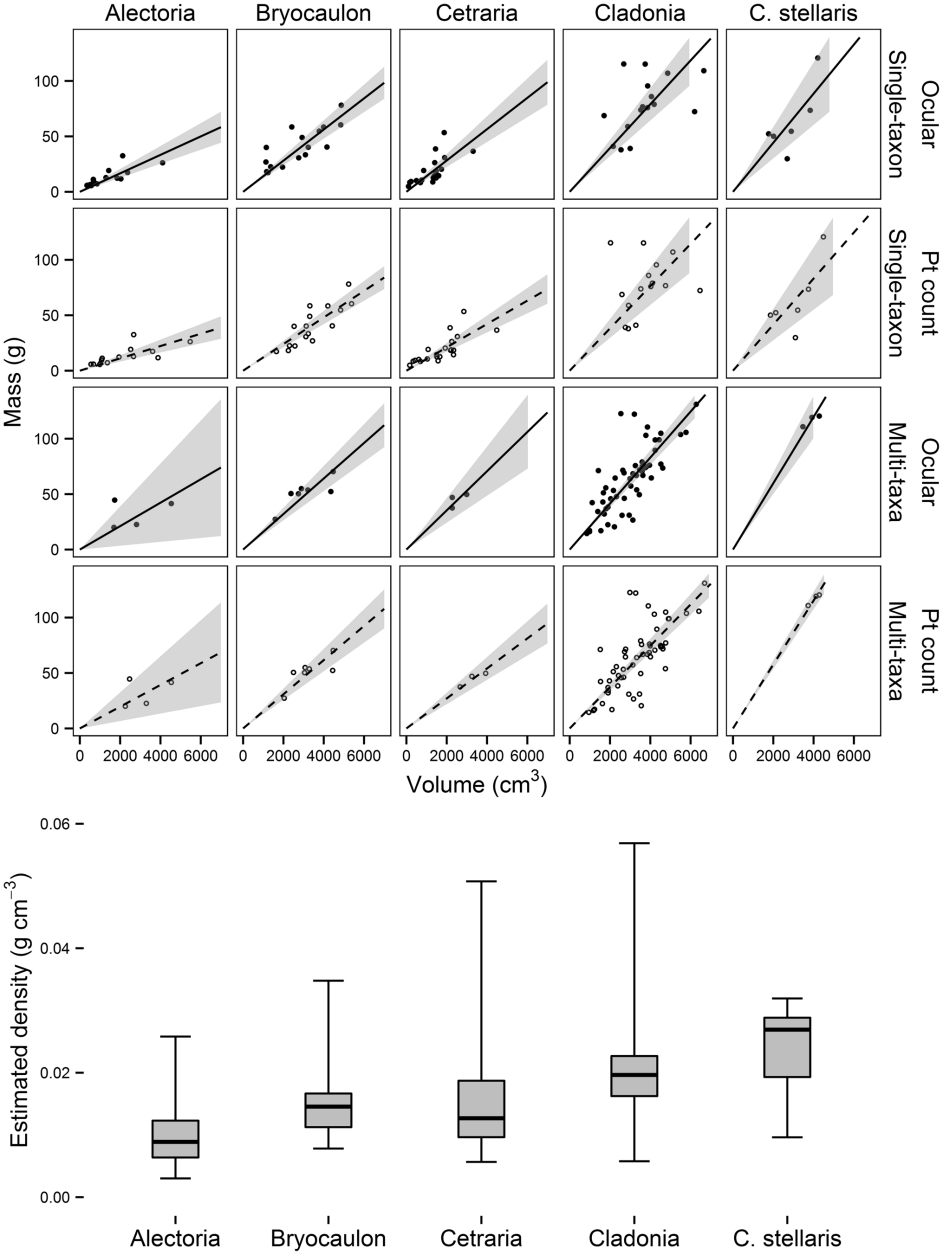
Biomass-volume relations and bulk density distributions for dominant forage lichen taxa in northwestern Alaska. Regressions were fitted using either single-taxon sampling (top two rows) or multi-taxa samples (bottom two rows). Lines indicate fitted model slopes (see Table 2 for estimates), while grey polygons indicate 95% confidence intervals. In the bottom panel, the distribution of estimated bulk density (all methods, grey boxes) for 144 lichen samples from northwestern Alaska is shown, where dark bars in boxes are median values, boxes represent the interquartile range of data values, and whiskers are the maxima/minima within each group. Mean bulk density differs among all species groups (*F*-test *p*<0.0001) except the pairwise comparisons among *Bryoria – Cetraria* and *Cladonia – C. stellaris* (for all others, *p*<0.05 or less from Tukey HSD test).

The point count method had lower biomass estimates relative to ocular methods (Fig. 1, filled vs. open symbols). This is directly related to the point count method estimating higher volume (Fig. 2A) and lower bulk density (Fig. 2B) relative to the ocular method. Model fit was usually but not always better with the ocular method compared to the point count method (Table 2). Among individual taxa, differences in slope between methods (Fig. 3) were more pronounced for samples dominated by taxa with diffuse, filamentous growth forms like *Alectoria*, rather than for denser taxa like *Cladonia* that tended to fill entire sample frames.

### Ecoregions and Landcover Types

When slope estimates were used to predict standing biomass for each of the four landcover types, the point count method had generally greater deviation from observed values than the ocular method (Table 3), though this was not true in all cases; magnitudes of deviation ranged from 0.01–19.5%. The biomass values reported here for the four landcover classes studied represents the high end of the lichen biomass distribution for each cover type because sites were selected based on high lichen cover to allow for better biomass-to-volume estimates. Of the seven combinations of landcover types nested within ecoregions (Table 1), there were 21 possible pairwise comparisons. Of the 21 comparisons, Tukey tests revealed very few significant pairwise differences for observed mass (3 significant comparisons), mass predicted using the ocular method (3 comparisons), mass predicted using the point count method (2 comparisons), and mass predicted using only height (0 comparisons). Each of these nominally “significant” pairwise comparisons had *p*-values that were close to the *p* = 0.05 significance threshold, so we do not report them here.

### Biomass vs. Height or Cover

Deviation of estimated values from observed values (Table 3) was similar but of slightly greater magnitude when using height as the sole predictor of biomass in multi-taxa communities than when using volume; magnitudes of deviation ranged from 0.5–18.1% for different landcover types. When using cover as the sole predictor of biomass, the deviation of estimated from observed values was far greater than when using volume (about 3–5 times greater: Table 3); this was true of the ocular method (absolute deviation: 3.1–28.4%) as well as the point count method (absolute deviation: 8.0–31.7%).

## Discussion

### Biomass as a Function of Volume

When lichen biomass is regressed on volume, the resulting linear slope is equivalent to a mass-to-volume ratio or a measure of the lichen mat's bulk density. Our findings indicate that taxa have different bulk densities as a result of their different growth forms, a trend that is reflected in multi-taxa communities dominated by each growth form. For individual taxa, *Cladonia* was most dense, while *Bryocaulon*, *Cetraria*, and *Alectoria* were successively less dense. There were congruent patterns in multi-taxa communities dominated by each of those genera, with *Cladonia*-dominated communities being densest.

The study most comparable with our methods [20] used volumetric estimation of biomass for four species (three *Cladonia* spp. and *Cetraria islandica*) in Sweden using 50×50 cm plots. Their mean *Cladonia* bulk densities agree closely with our estimates (within 12% of each other for ocular methods), but were slightly lower, perhaps because they manually pulled lichen mats off the ground (rather than cut them cleanly as we did), which could leave lichen material attached to the ground. By contrast, *Cetraria* spp. bulk densities from our study were nearly 50% less than that of *C. islandica* in Sweden [20]. We assume that unmeasured factors (e.g., nutrient availability, moisture, growing season, fire regime, successional status, herbivory patterns) allow *Cetraria* in Sweden to occur in much denser colonies than on the Seward Peninsula of Alaska.

In Finland, Kumpula et al. [2], reported a much lower bulk density than either our results or those from Sweden [20], possibly resulting from differing definitions of what constitutes a “living” portion of a lichen thallus. Dunford et al. [5] did not report a bulk density because they relied solely on lichen cover (not height or volume) for the single species *Cladonia mitis*. Though their scope of inference extended to 25 sites, Dunford et al. [5] also had a drastically smaller sample size than ours (*n* = 8 vs.144).

To the extent that we had outlier observations, these were likely due to the unintentional inclusion of non-target vegetation in some plots, especially where lichen thalli were layered over top of unseen moss or shrub tissues beneath. While our volumetric calculations probably included portions of non-target vegetation, we note that this is consistent with all situations in which non-destructive estimates of cover and height are used for applying conversion factors to larger study areas.

### Single-taxon vs. Multi-taxa Sampling

Individual genera had generally lower bulk density than the multi-taxa samples. This is probably because lichens on the Seward Peninsula grew in mixed assemblages with intertwined and overlapping layers composed of different growth forms and bulk densities. Thus what superficially appeared to be a continuous cover of one lichen from the surface was in fact usually a tangled mat of several different lichen taxa below. This may be why we found it difficult to locate monotypic mats for every target species, even using a plot size of just 25×25 cm.

Estimating biomass–volume ratios for every species found in northwestern Alaska would be a prohibitive task. Given that multi-taxa surveys require only general taxonomic training and that they capture variation in biomass at levels equivalent to or better than individualistic single-taxon assessments, we conclude that a multi-taxa focus is preferable to single-taxon surveys when lichens across entire landscapes must be faithfully represented.

### Ocular vs. Point Count

The use of point-count estimations of cover gave consistently higher cover (and volume) estimates than ocular methods, leading to lower bulk density estimates; this is consistent with other findings [20]. Despite its initial appeal for reducing observer bias, we noted that the point count method often seemed to overestimate cover by “catching” or touching disproportionately small pieces of lichen thallus in areas of the plot that lacked appreciable coverage, especially for taxa that were filamentous (e.g., *Alectoria*) or patchily distributed. Though the ocular method seemed initially less objective, it had the benefit that it allowed observers to visually integrate patchy or diffuse spatial coverage of lichens in a more realistic manner. Point count methods overestimate cover of vascular plants, while visual estimates are more accurate [27], and counting enough points to be useful would be logistically limiting in remote Arctic settings. We note that point count methods may still have utility for plots larger than our 25×25 cm size because ocular cover becomes less precise in larger plots [15]. Point count methods may also be preferable for long-term monitoring where repeatability is paramount [28] and where results may differ among observers [14]. Future work is needed to resolve variation among observers and identify sources of error.

### Ecoregions and Landcover Types

The two ecoregions we examined on the Seward Peninsula appeared to have similar bulk densities (g cm^−3^) of lichens. Bulk densities also differed far less than expected among the four landcover types. We expected that differences in substrate availability, substrate chemistry, moisture and vascular vegetation would be expressed in lichen community biomass because growth forms differ according to those conditions. For example, filamentous groups such as *Bryocaulon* and *Alectoria* dominate the Sparse Vegetation (“S”) cover type on the Seward Peninsula, whereas the low-elevation L, P, and M landcover types tend to be dominated by denser *Cladonia* spp. We suspect that ecoregion and landcover would have been more influential had we sampled across a broader latitudinal gradient (see [29] for one example of an Arctic gradient spanning 1800-km north-south). Note that while the biomass values in Table 3 are a starting point for landscape modeling, they portray only one possible scenario (i.e., one with lichen cover averaging ∼80% due to our preferential plot assignment). Future landscape estimates must account for patchy or sparse lichen cover, and therefore require a truly random sample of sites from within each landcover type.

### Biomass vs. Height or Cover

In some instances, a direct measure of biomass as a function of average height of the lichen can be useful, avoiding the need to estimate cover. For example, line intercept methods can provide copious amounts of height data in a short time frame and may be more applicable than closed-frame methods in the open, unforested landcover types that are commonly found in northwestern Alaska. However, we found a large amount of variation in the estimates that relied on height as the sole predictor, and we advise that height-based estimates must be always weighted by some measure of cover to prevent overestimation. Given that caveat, transect-based line intercept methods could provide biomass estimates if the total area of the study site is known.

If height alone appeared to be an inadequate predictor of lichen biomass, then cover alone was even worse. This makes logical sense – two different lichen patches could cover an equal surface area yet differ by an order of magnitude in height; observers would have no way of knowing their true volume. Because we found that deviations from true values were 3 to 5 times greater when using cover alone as compared to the other methods, we do not recommend using cover as the sole predictor of lichen biomass.

### Extending Spatial Coverage

Remote sensing is one way that estimates can be scaled up to landscape-level inferences. Field surveys can be coupled with remote sensing imagery to construct biomass estimates via several algorithms [4]. Other workers [30] have similarly used Landsat imagery to derive leaf area index and biomass for multiple vegetation groups including fruticose, foliose and crustose lichens (in aggregate). Spectral decomposition of remote imagery is a further refinement that can distinguish among taxonomic or functional groups of lichens [31]. Though location-specific correction factors must always be derived and validated on the ground, remote sensing is a promising technology that should allow rapid estimation of biomass for very large, remote or difficult-to-access locations in northern ecosystems.

### Management Applications

The biomass functions presented here promise to have utility in several different areas of applied ecology in arctic Alaska. Studies of ungulate biology should be able to use these equations to help scale plot-level vegetation data (where both lichen cover and height measurements are available) to remote sensing imagery, as in [32]. Long-term studies of ungulate grazing exclosures are underway on Alaska's Seward Peninsula [33], [34], and conversion of lichen cover/height data to forage biomass will be an essential component of these studies. The National Park Service has other long-term lichen/vegetation studies in which both lichen cover and height are measured [33]; these may similarly be coupled with remote sensing modeling using our conversion factors for applications including changes in vegetation community structure with climate change effects [35], vegetation response to changing fire regimes [36], and carbon accounting [37]. The National Park Service's Arctic Network has sufficient density of ground-based measurements to model lichen biomass at a landscape scale [24]. Repeat measurements would allow for temporal and spatial depictions of lichen biomass on the landscape. Improved burn severity geospatial layers and historic fire perimeters would increase the resolution of these layers by allowing for the modeling of successional status, and hence lichen height and cover. Lichen winter range for the Western Arctic Caribou Herd is projected to suffer some decline with climate-driven shrub increase [1], [35], more frequent wildfire [36], and increasing inputs of nitrogen and sulfur from regional development [38]. Continued monitoring of lichen biomass will be critical for detecting and addressing ongoing changes.

## Conclusions

To summarize, we found that multi-taxa (“bulk”) sampling, when coupled with ocular estimation, was the preferred method for biomass estimation because it yielded the most accurate estimates and was logistically most efficient in the field. This method required no more than general taxonomic knowledge in the field and was tractable for statistical purposes. The zero-intercept regression equation (biomass = 0.0203 g cm^−3^) which we derived by this method is readily applied to lichen communities in northwestern and arctic Alaska, though we caution that this does not necessarily apply to other geographical regions where location-specific equations will be required. Height alone was not the preferred predictor of biomass, and cover alone was a poor predictor; the best estimates should ideally include both height and cover. Because bulk densities varied among the forage lichen species we measured, we suggest that in cases where there is only a single species of interest, investigators may benefit from using separate regression equations for target species. Lichen biomass estimation has a wealth of applications that will help managers estimate wildlife forage, understand successional trends, detect climate and air quality signals, and account for landscape-level carbon. Therefore, continued monitoring will be vital for understanding how ongoing changes in lichen biomass and distribution affect other elements of Arctic ecosystems.

## Supporting Information

Dataset S1 **Cover, height and biomass of terrestrial lichens in northwestern Alaska.**
(CSV)
